# Editorial: The “proteine 2022” conference

**DOI:** 10.3389/fmolb.2023.1157442

**Published:** 2023-02-22

**Authors:** Francesco Balestri, Adeline Goulet, Gabriella Tedeschi

**Affiliations:** ^1^ Department of Biology, Biochemistry Unit—University of Pisa, Pisa, Italy; ^2^ Laboratoire d'Ingénierie des Systémes Macromoléculaires, LISM UMR7255 CNRS & Aix-Marseille Université, Marseille, France; ^3^ Department of Veterinary Medicine and Animal Science—University of Milano, Lodi, Italy

**Keywords:** proteins, biochemistry, proteins annual meeting, Italian society of biochemistry, proteins group

The Research Topic of this Research Topic aims to gather the Proceedings of “Proteine 2022” meeting (Pisa, May-18-20) organized by the Protein Group of the Italian Society of Biochemistry (SIB). This Research Topic is focused on protein-protein and protein-ligand interactions in order to obtain new relevant knowledge about the discovery, design and the rational development of drugs for the treatment of particular diseases.

In this Research Topic new important data regarding the Coronavirus Disease 2019 (COVID-19) caused by the severe acute respiratory syndrome coronavirus 2 (SARS-Cov-2) are presented. The manuscript of Iacobucci et al., focuses on the viral glycoprotein Spike S which is involved in the recognition and interaction of viral particles with surface host receptors as well as in the fusion of the viral capsid with the host cell membrane. Authors investigated the Spike S1 subunit interactomes looking for potential additional Spike human receptors that could possibly be involved in other intracellular processes in non-pulmonary systems. Shotgun proteomics approaches were used for protein identification and several bioinformatics approaches were performed for functional data analysis. The proteomic experiments allowed the authors to identify many host protein targets and ascertain the role of the S1 domain in all steps of the interaction between the pathogen and the different host cell lines used in this research. Their data suggest the role of S1 not only in receptor recognition but also in vesicle formation; an interplay between S1 domain and human proteins associated with energetic cell metabolism in almost all analysed cell lines has been highlighted. In the manuscript of Dos Santos et al., Authors showed a strong correlation between enhanced in vivo proteolysis and mortality in patients with septic shock caused by a bacterial infection as well as by COVID-19-induced bacterial superinfection. They used a peptidomic-based approach in order to estimate the magnitude of *in vivo* proteolysis by assessing the abundance and count of peptides in the plasma sample. The analysis of the circulating proteome and peptidome was reinforced by the quantitation of the proteolytic activity of proteases that are activated during the acute form of the disease in order to obtain more information on the role of proteases in the regulation of the balance between coagulation and fibrinolysis in the systemic intravascular proteolysis observed during acute illness. The correlation between proteolytic activity and protease expression patterns in plasma during bacterial superinfection can provide pathophysiologically and clinically valuable information that could be useful to anticipate the signs of possible coagulation disorders and aid in early diagnosis providing useful devices for a timely therapy.

This Research Topic also reports data on protein-protein and protein-ligand interactions related to the search for antibacterial drugs and the understanding of key cellular signaling pathways in cell migration.

The manuscript of Kabongo et al., deals with the drug discovery related to the enzyme malate:quinone oxidoreductase (MQO), a peripheral membrane protein essential for the survival of several bacteria and parasites such as *C. jejuni* which is the most common cause of bacterial foodborne diseases worldwide. The increasing incidence and antibiotic resistance of this bacterial infection require the adoption of new strategies in order to counteract the infection spread. The MQO enzyme catalyses the oxidation of malate to oxaloacetate and is also involved in the reduction of the quinone pool in the electron transport chain thereby contributing to cellular bioenergetics. For these reasons the enzyme is an attractive drug target as it is not conserved in mammals. Authors optimized the overexpression and purification of MQO from *Campylobacter jejuni* (CjMQO), conducted an optimization of CjMQO assay conditions with a determination of enzyme steady-state kinetic parameters and reaction mechanism and finally, investigated the inhibition mechanism of two CjMQO inhibitors of plant origin, ferulenol and embelin. These molecules strongly inhibit the CjMQO enzymatic activity as well as the growth of *C. jejuni*, and hence offer promising perspectives as an antibacterial tool.

Lastly, the paper of Vacchini et al. applies a quantitative SILAC-based phosphoproteomic analysis coupled to a systems biology approach with network analysis to investigate the signaling pathways downstream to ACKR2, an atypical chemokine receptor which is structurally uncoupled from G proteins and is unable to activate signaling pathways used by conventional chemokine receptors to promote cell migration. ACKR2 regulates inflammatory and immune responses by shaping chemokine gradients in tissues *via* scavenging inflammatory chemokines. The analysis was carried out on a HEK293 cell model expressing either ACKR2 or its conventional counterpart CCR5. The model was stimulated with the common agonist CCL3L1 for short (3 min) and long (30 min) durations. As expected, many of the identified proteins are known to participate in conventional signal transduction pathways and in the regulation of cytoskeleton dynamics. However, the analyses revealed unique phosphorylation and network signatures, suggesting roles for ACKR2 other than its scavenger activity providing an unprecedented level of detail in chemokine receptor signaling and identifying potential targets for the regulation of ACKR2 and CCR5 function.

In addition to the contributions to the Research Topic, the Congress itself had a good participation offering an overview of the state of the art of Italian research in the field of proteins.

Furthermore, we were particularly pleased to see the significant participation of many of the younger generation scientists presenting their work both as posters and in oral communications.

